# Microevolution of Monophasic *Salmonella* Typhimurium during Epidemic, United Kingdom, 2005–2010

**DOI:** 10.3201/eid2204.150531

**Published:** 2016-04

**Authors:** Liljana Petrovska, Alison E. Mather, Manal AbuOun, Priscilla Branchu, Simon R. Harris, Thomas Connor, K.L. Hopkins, A. Underwood, Antonia A. Lettini, Andrew Page, Mary Bagnall, John Wain, Julian Parkhill, Gordon Dougan, Robert Davies, Robert A. Kingsley

**Affiliations:** Animal and Plant Health Agency, Addlestone, UK (L. Petrovska, M. AbuOun, M. Bagnall, R. Davies);; The Wellcome Trust Sanger Institute, Cambridge, UK (A.E. Mather, S.R. Harris, T. Connor, A. Page, J. Parkhill, G. Dougan, R.A. Kingsley);; Institute of Food Research, Norwich, UK (P. Branchu, R.A. Kingsley);; Public Health England, London, UK (K.L. Hopkins, A. Underwood, J. Wain);; Istituto Zooprofilattico Sperimentale delle Venezie, Legnaro (PD), Italy (A.A. Lettini)

**Keywords:** Salmonella, molecular epidemiology, monophasic, microevolution, heavy metal resistance, MDR, Salmonella Typhimurium, bacteria, United Kingdom, antimicrobial resistance

## Abstract

Microevolution associated with emergence and expansion of new epidemic clones of bacterial pathogens holds the key to epidemiologic success. To determine microevolution associated with monophasic *Salmonella* Typhimurium during an epidemic, we performed comparative whole-genome sequencing and phylogenomic analysis of isolates from the United Kingdom and Italy during 2005–2012. These isolates formed a single clade distinct from recent monophasic epidemic clones previously described from North America and Spain. The UK monophasic epidemic clones showed a novel genomic island encoding resistance to heavy metals and a composite transposon encoding antimicrobial drug resistance genes not present in other *Salmonella* Typhimurium isolates, which may have contributed to epidemiologic success. A remarkable amount of genotypic variation accumulated during clonal expansion that occurred during the epidemic, including multiple independent acquisitions of a novel prophage carrying the *sopE* gene and multiple deletion events affecting the phase II flagellin locus. This high level of microevolution may affect antigenicity, pathogenicity, and transmission.

## Abstract 2

Microevolution resulted in considerable genotypic variation.

*Salmonella enterica* is one of the most common enteric pathogens of humans and animals. An estimated 94 million cases of nontyphoidal salmonellosis occur worldwide each year, causing considerable illness and death; in the United States, the associated economic burden estimated by the US Centers for Disease Control and Prevention is >$2 billion US per year (*1*,*2*). 

*S. enterica* consists of >2,500 serovars, of which *S*. *enterica* serovar Typhimurium (*Salmonella* Typhimurium) is the most ubiquitous in zoonotic reservoirs for human infection and the environment (*3*). Over the past half century, the epidemiology of *Salmonella* Typhimurium has been characterized by successive waves of dominant multidrug-resistant clones (*4*). During 1966–2010 in Europe, where variants are distinguished by definitive (phage) type (DT), *Salmonella* Typhimurium DT9, DT204, DT104, and DT193 emerged successively as multidrug-resistant strains (*5*). Epidemic strains dominate for 4–15 years before being replaced by a new dominant phage type. The emergence and spread of *Salmonella* Typhimurium DT104 was global (*6*) and largely responsible for the increased multidrug-resistant *Salmonella* isolates in Europe and North America in the 1990s (*7*). As DT104 incidence has waned in the United Kingdom, monophasic variants of *Salmonella* Typhimurium with the antigenic formula 1,4,[5],12:i:- have emerged (*8*), although it is not clear if this current monophasic *Salmonella* Typhimurium epidemic is related to other epidemics of monophasic variants previously reported in North America (*9*), Spain (*10*), and elsewhere in Europe (*11*). Analysis of the genomic deletions in the phase II flagellum locus responsible for the monophasic phenotype suggested that multiple independent clones may be emerging in the United States and Europe (*9*). 

The first description of a monophasic *Salmonella* Typhimurium epidemic in Europe was that of a “Spanish clone,” which emerged rapidly during 1997 and was characterized by a deletion in the allantoin–glyoxylate operon and the *fljAB* operon, phage type U302, and a heptaresistance pattern ACSuGSTSxT (resistant to ampicillin, chloramphenicol, sulfonamide, gentamicin, streptomycin, tetracycline, and co-trimoxazole) (*10*). Since this time, many European countries have reported increased incidence of this serotype, particularly associated with pig herds (*12*–*15*) but later with cattle (*16*,*17*). However, in contrast to the Spanish clone, these current monophasic *Salmonella* Typhimurium epidemic strains have commonly been associated with phage types DT193 or DT120 and a predominant ASSuT tetraresistance pattern (resistant to ampicillin, streptomycin, sulfonamide, and tetracycline), suggesting that the epidemics are distinct.

The molecular basis for the success of epidemic clones of bacterial pathogens has implications for the surveillance and management of infectious diseases. Epidemiologic success depends on selective advantage of epidemic clones, resulting from their unique genotype. The current multidrug-resistant *Salmonella* 4,[5],12:i:- epidemic in the Europe was first reported around 2005 and is mainly associated with isolates of phage types DT193 and DT120 (*18*). 

We investigated the phylogenetic relationship of 206 strains of *Salmonella* Typhimurium (*Salmonella* 1,4,5:i:1,2) and monophasic *Salmonella* Typhimurium (*Salmonella* 1,4,[5],12:i:-), isolated from humans, livestock, or contaminated food from the United Kingdom or Italy from 1993 through 2010. We report the whole-genome sequence variation of *Salmonella* Typhimurium and *Salmonella* 1, 4,[5],12:i:- isolates from the United Kingdom and Italy and the application of these data to phylogenetic reconstruction of the epidemic. We address the questions of whether the monophasic *Salmonella* Typhimurium isolates in the United Kingdom are part of a single epidemic and how they are related to previously circulating biphasic and monophasic *Salmonella* Typhimurium strains. 

## Materials and Methods

We used bacterial isolates from strain collections held by the Animal and Plant Health Agency (Addlestone, UK); Public Health England (Colindale, London, UK); or the National Regional Laboratory for *Salmonella,* Istituto Zooprofilattico Sperimentale delle Venezie (Legnaro, Italy). The serotype and phage type were determined as previously described (*19*). The presence of the *fljB* locus and the occupancy of the *thrW* locus was initially determined by PCR amplification as previously described (*11*). Strain selection was intended to represent the diversity of *Salmonella* Typhimurium in the United Kingdom and not to be representative of the epidemiology (Technical Appendix 1). 

To determine antimicrobial drug sensitivity, we tested isolates from animals in the United Kingdom and Italy for susceptibility to antimicrobial drugs according to standard procedure (*20*). Resistance or susceptibility were interpreted on the basis of British Society for Antimicrobial Chemotherapy break points; we report the intermediate category as resistant. We determined antimicrobial drug sensitivity of isolates from human patients in the United Kingdom by using a modified break-point technique on Iso-Sensitest agar (Oxoid, Basingstoke, UK) (Technical Appendix 2). The MIC for copper sulfate was the concentration at which bacterial growth optical density 600 nm was >0.1 after culture (without shaking) at 37°C for 24 hours in Luria Bertani (Oxoid) broth buffered with 25 mmol/L HEPES (4-[2-hydroxyethyl]-1-piperazineethanesulfonic acid) at pH7. We then determined the whole-genome sequence by using the HiSeq Illumina (http://www.illumina.com) platform, sequence analysis, de novo assembly, annotation, and PCR amplification (Technical Appendix 2).

## Results

### *Salmonella* 4,[5],12:i:- Strains

**Figure 1:**
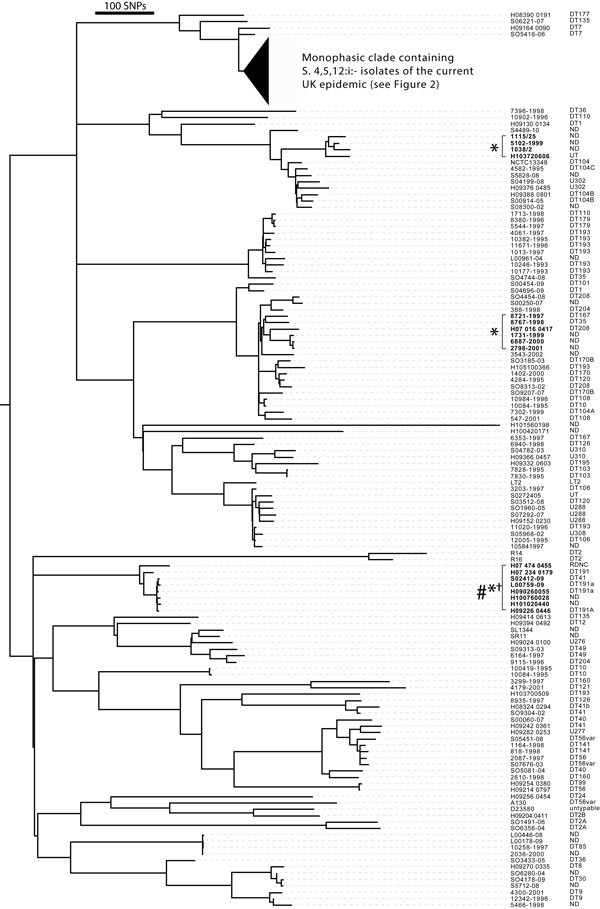
Phylogeny of *Salmonella enterica* serovar Typhimurium (*Salmonella* Typhimurium) and *Salmonella* 1,4,[5],12:i:- isolates from the United Kingdom and Italy, 2005–2010. Maximum-likelihood tree of 212 *Salmonella* Typhimurium and monophasic isolates was constructed by using 12,793 single-nucleotide polymorphisms (SNPs) outside of prophage elements, insertion sequence elements and sequence repeats identified by reference to the whole-genome sequence of *Salmonella* Typhimurium strain SL1344. The tree is rooted with *Salmonella* Enteritidis whole-genome sequence as an outgroup (note shown). The lineage containing the *Salmonella* 1,4,[5],12:i:- current UK epidemic group is conflated for simplicity (filled triangle). The designation of the isolates (left column) and phage type are shown (right column). *Monophasic isolates outside of the main epidemic clade. †Monophasic clade closely related to the monophasic clone CVM23701 from North America (*9*). DT, definitive (phage) type; ND, not determined. Scale bar indicates the approximate number of SNPs determined by genetic distance and the number of SNPs used to construct the tree.

We determined that contemporary *Salmonella* 4,[5],12:i:- strains in the United Kingdom are part of a single clonally expanding clade. We constructed a maximum-likelihood phylogeny of all 97 monophasic and 142 *Salmonella* Typhimurium strains (Technical Appendix 1 Table) by using 12,793 variable sites in the genome, with reference to the whole-genome sequence of reference strain SL1344, excluding single-nucleotide polymorphisms (SNPs) in prophage, insertion sequence elements, and repetitive sequences (Figure 1). Most (77 of 97) monophasic strains were from a single distinct clade that seemed to be part of the current monophasic *Salmonella* Typhimurium epidemic because they were the most abundant and most recently isolated strains. However, older monophasic isolates were also found in at least 3 other clades within the *Salmonella* Typhimurium tree (Figure 1, indicated with *). A clade containing 8 isolates including 2 DT191a (Figure 1, indicated with†) was closely related to a *Salmonella* 1,4,[5],12:i:- isolate from the North American epidemic strain CVM23701 (*9*). Only 6 SNPs distinguished this isolate from strain H07 474 0455. In addition, a clade containing 6 *Salmonella* Typhimurium var. Copenhagen (4,12:i:1,2) strains (e.g., H070160417) and a clade containing 4 isolates (e.g., H103720606) contained monophasic strains.

### Phylogenetics of Monophasic *Salmonella* Typhimurium 

**Figure 2:**
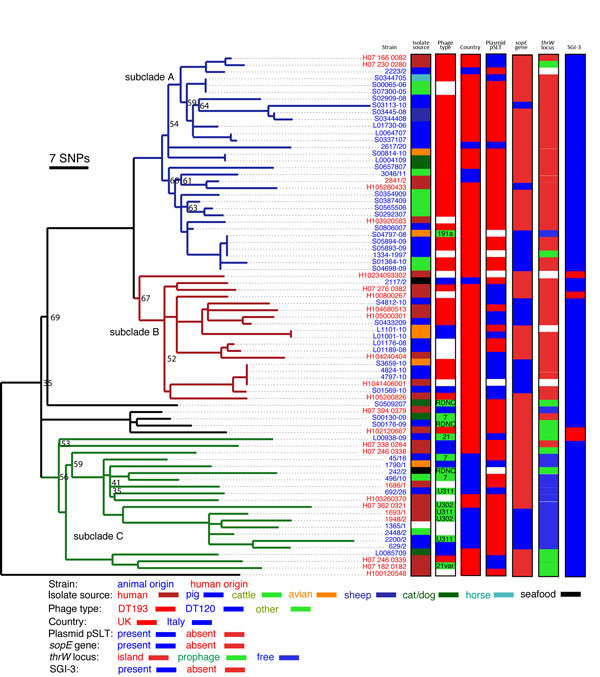
Phylogeny of *Salmonella* 1,4,[5],12:i:- epidemic clade isolates from the United Kingdom and Italy, 1993–2010. Maximum-likelihood tree of 77 *Salmonella* 1,4,[5],12:i:- isolates rooted with *Salmonella* Typhimurium strain SL1344 was constructed by using 1,058 single-nucleotide polymorphisms (SNPs) outside of prophage elements, insertion sequence elements, and sequence repeats identified with reference to whole-genome sequence of *Salmonella* Typhimurium strain SO4698-09. Bootstrap values <70 are indicated at nodes. Subclades A (blue lineages), B (red lineages), and C (green lineages) are indicated. Strain designations are color coded for isolates from humans (red) and animals (blue). Epidemiologic data for the source of isolate, phage type, country of origin, presence of the virulence plasmid (pSLT), presence of the *sopE* gene, occupancy of the *thrW* locus, and presence of *Salmonella* genetic island 3 are indicated (right). Scale bar indicates the approximate number of SNPs determined by genetic distance and the number of SNPs used to construct the tree.

A maximum-likelihood phylogenetic tree, reconstructed by using variable sites within the whole-genome sequence with reference to the draft genome sequence of a representative strain from within the epidemic (strain SO4698-09), indicated a clonally expanding clade with a maximum root-to-tip distance of ≈70 SNPs. This finding indicated that all strains in the tree shared a common ancestor in the recent past (Figure 2). All isolates from this monophasic clade were of sequence type 34. The phage type of monophasic epidemic isolates varied according to phylogeny. Most isolates were DT193 (38 of 62 typed) or DT120 (*9*) and various other phage types including DT7 (*3*), DT191a (*1*), DT21 (*1*), DT21var (*1*), U311 (*3*), U302 (*2*), and RDNC (*3*). However, although virtually all isolates in subclades A and B were DT193, the phage type was highly variable in subclade C. Biphasic DT193 strains (e.g., 4061-1997; Figure 1) isolated before 2005 were not direct ancestors of the current monophasic *Salmonella* Typhimurium epidemic because they were present on a distinct lineage. Indeed, DT193 isolates were present on 4 distinct lineages within the phylogenetic tree, highlighting the polyphyletic nature of this phage type (Figure 1). Isolates from UK animals in subclade C were relatively scarce; 1 of 21 isolates in this subclade was from a UK animal. Instead, isolates from this subclade came predominantly from humans in the United Kingdom and humans and animals in Italy. In contrast, isolates from subclade A were mostly (18 of 32) of livestock origin; only 5 were of human origin. Clade B contained an approximately equal number of human and livestock isolates. Furthermore, although isolates from UK pigs were present in all 3 subclades, isolates from UK cattle were present only in subclade A, consistent with epidemiologic reports that the epidemic originated in pig herds and later spread to cattle herds (*17*). Despite analysis inclusion of only 6 isolates from birds, these were distributed throughout the tree, suggesting multiple transmission events into these animal populations. The distribution of isolates from humans and livestock (pigs, cattle, and sheep) within subclades of the phylogenetic tree of UK monophasic isolates was also strikingly uneven. Most (64 of 77) isolates were ASSuT tetraresistant, and the corresponding resistance genes were detected in de novo assembled sequences (Technical Appendix 2 Figure 1), suggesting that the most recent common ancestor (MRCA) of the clade had this complement of resistance genes. However, during clonal expansion, 7 strains had lost their resistance genes entirely and another 7 had an altered complement of resistance genes.

### Novel Genetic Island Encoding Resistance to Heavy Metals

**Figure 3:**
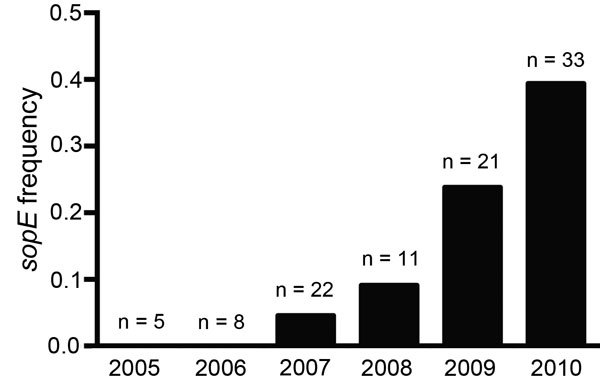
Frequency (proportion) of carriage of the *sopE* gene in *Salmonella* 1,4,[5],12:i:- epidemic isolates from the United Kingdom and Italy for each year during 2005–2010. The presence of the *sopE* gene was detected in draft genome assemblies by sequence comparison or by PCR amplification of genomic DNA by using primers specific for the *sopE* gene of randomly selected monophasic isolates from each year. The number of isolates investigated for each year is indicated above the bar.

A large novel genomic island (designated SGI-3) specific to the monophasic *Salmonella* Typhimurium epidemic clade is inserted at the *yjdC* locus (Technical Appendix 2 Figure 2) in strain SO4698-09. The island contained ≈90 genes, some of which had sequences similar to those associated with plasmid transfer and conjugation, and an integrase gene, suggesting that the island may have originated by integration of a plasmid. Determination of the accessory genome indicated that the island was present in 74 of 77 isolates within the monophasic clade (Figure 2) but was absent from all strains from outside the clade. Ancestral state reconstruction performed by using ACCTRAN (*21*) (Technical Appendix 2 Figure 3, panel A) suggested that this island was probably introduced shortly before clonal expansion of the monophasic clade. Three clusters of genes similar to genes involved in resistance to heavy metals are present on the island. Consistent with the island contributing to enhanced resistance to copper sulfate, a common animal feed additive, the MIC (p = 0.015) for copper sulfate was significantly greater for isolates within the monophasic *Salmonella* Typhimurium clade (24.2 ± 1.9 mmol/L) than for *Salmonella* Typhimurium isolates from outside this clade (21.2 ± 1.1 mmol/L) that did not encode the island (Technical Appendix 2 Figure 4).

### Genotypic Variation in the *fljBA* and *thrW* Loci and Loss of the Virulence Plasmid

The monophasic phenotype results from the absence of phase-2 flagellin monomer FljB. The presence of the *fljBA* genes and the neighboring genome sequence of *Salmonella* Typhimurium and monophasic variants, determined by mapping raw sequence read data to the *fljB* locus region of the SL1344 whole-genome sequence (Technical Appendix 2 Figure 5, panel A), indicated that the UK epidemic strains are monophasic because of multiple independent deletion events that occurred during clonal expansion. Four *Salmonella* Typhimurium isolates (2 DT7 isolates [SO5416–06 and H09164 0090], 1 DT135 isolate [SO6221–07], and 1 DT177 isolate [H08390 0191]) that were closely related and shared a common ancestor with the monophasic epidemic strains (Figure 1) encoded the entire *fljBA* locus, indicating that the MRCA with these strains and the epidemic strains was biphasic. In contrast, 67 of 77 monophasic *Salmonella* Typhimurium strains from the epidemic clade lacked at least part of the *fljBA* locus, resulting from deletions ranging in size and with a distribution that was consistent with the phylogenetic relationship of the strains (Technical Appendix 2 Figure 5, panel A). The 8 epidemic strains that did not have a deletion in the *fljB* locus were deeply rooted in the tree, consistent with multiple deletion events (1–36 kb) occurring since clonal expansion of the clade. Most deletions shared a common junction in the intergenic region of *fljB* and *iroB*. Because it was not possible to assemble short read sequence data across the *fljB* locus deletion region, to investigate the nature of the deletion, we generated long read sequence data for a representative isolate SO4698–09 by using the PacBio sequencing platform (Pacific Biosciences, Menlo Park, CA, USA). A single contig assembly of these data revealed a 15,726-bp deletion of the genome relative to SL1344 and a 27,473-bp insertion of a novel sequence (Technical Appendix 2 Figure 5, panel B). The inserted sequence was similar to sequences of several genes from transposon Tn21, mercury resistance genes (*merTABCDE* and *merR*), and antimicrobial drug resistance genes, consistent with the resistance profile of this strain (*strA*, *strB*, *sul2*, *tet[B]*, and *bla*_TEM-1_). The composite transposon insertion was not present in closely related isolates (e.g., SO5416-06) (Figure 1) that were outside of the monophasic clade, suggesting that it was acquired by the MRCA of the monophasic clade and not before clonal expansion. The deletions in the *fljB* locus of monophasic strains from outside the main clade from the United Kingdom were distinct from that in the UK monophasic clade but identical to those described for strains from epidemics in North America (e.g., CVM23701) (*9*) and Spain (e.g., 1115/25) (*10*) (Technical Appendix 2 Figure 5, panel A).

In addition to hypervariability at the *fljB* locus, isolates from the epidemic group exhibited sporadic loss of the virulence plasmid pSLT. The pattern of plasmid loss within the clade could be most parsimoniously explained by loss during clonal expansion. Of note, the loss of pSLT was not uniform across the monophasic tree. Although only 13% and 20% of isolates tested contained pSLT in subclades A and C, respectively, in contrast, >70% of isolates in subclade B contained the plasmid (Figure 2).

### *sopE* Virulence Gene 

The *sopE* virulence gene was acquired on a novel prophage, mTmV (monophasic *Salmonella* Typhimurium V), by multiple independent events during clonal expansion of the epidemic clade. The *thrW* locus of contemporary monophasic *Salmonella* Typhimurium isolates has been reported to harbor either a prophage, a novel genetic island, or neither (*11*). In strain SO4698-09, the *thrW* locus contains the novel genetic island described previously but also an additional prophage element encoding the *sopE* gene that together total 55 kb. Determination of the accessory genome by using the Roary pan genome pipeline (*22*) indicated that 23 of 77 monophasic isolates from the epidemic clade contained the *sopE* gene (Figure 2). SopE is a guanine exchange factor involved in subversion of the host enterocyte cytoskeleton, a key component of the infection process (*11*,*23*,*24*). The *sopE* gene was present in 6 distinct clusters of the monophasic clade, and ancestral state reconstruction indicated that multiple independent acquisitions followed by clonal expansion of the *sopE* -positive variant was the most likely explanation for their distribution (Technical Appendix 2 Figure 3, panel B). The *sopE* gene of strain SO4698-09 is present on a 55-kb region, designated mTmV phage, which was absent from strain SL1344 and shared the greatest similarity with the *Shigella flexneri* V prophage (Technical Appendix 2 Figure 6) (*25*). The mTmV phage from SO4698-09 was not related to the FELS-2 prophage of *Salmonella* Typhimurium strain SL1344, which also encodes the *sopE* gene, except in a 2,443-bp region that encoded the *sopE* gene and flanking sequence. Examination of partial assemblies of other monophasic strains encoding *sopE* revealed that the gene was associated with the same prophage and inserted between the genome region corresponding to the *thrW* locus. These data indicated that a novel *sopE* phage entered the genome on at least 6 occasions during the clonal expansion of the epidemic clade. Because the *sopE* gene was present in phylogenetic clusters toward the terminal branches of the monophasic clade tree and subsequently exhibited clonal expansion, we addressed the question of whether the proportion of strains that encoded the *sopE* gene in our strain collection each year changed during 2005–2010. The frequency distribution for each year was determined from collated data from 59 strains for which date of isolation and sequence data were available and an additional 41 randomly selected monophasic strains from the United Kingdom for which the presence of the *sopE* gene was determined by PCR (Figure 3; Technical Appendix 2 Table). Increased frequency, ranging from none in 2005 and 2006 to 40% in 2010, suggested that acquisition of this gene may have conferred a competitive advantage.

## Discussion

We identified a remarkable level of microevolution during clonal expansion of the epidemic. Such expansion may affect the antigenicity, pathogenicity, and transmission of monophasic *Salmonella* Typhimurium.

The phylogenetic relationships of *Salmonella* 1,4,[5],12:i:- isolated from the United States and Europe since the late 1990s is unclear from reports to date. Our analyses suggest that at least 3 distinct epidemics have been associated with *Salmonella* 1,4,[5],12:i:- and that most of the monophasic isolates from livestock and humans in the United Kingdom since 2006 are not directly related to isolates from either the epidemic in Spain around 1997 (*10*) or the epidemic in the United States around 2004 and 2007 (*9*). Instead, the UK epidemic is related to that reported in Germany and elsewhere since around 2005 (*11*). The US clone is characterized by a large deletion in the *fljB* locus and acquisition of a prophage, neither of which were present in the UK monophasic clone. Furthermore, the whole-genome sequence for a single isolate from the US epidemic (CVM23701) placed this isolate in a small clade of monophasic isolates from the United Kingdom isolated around 1995, distinct from the current UK clade. The clone from Spain is characterized by variable size deletions in the *fljB* locus, all distinct from deletions observed in the UK isolates, and a deletion in the allantoin metabolism locus, also absent from the main UK clade. The MRCA of the UK *Salmonella* 1,4,[5],12:i:- epidemic in our strain collection was shared with a biphasic *Salmonella* Typhimurium isolate with DT7 (strain H091640090), a relatively rare phage type that has not been associated with epidemics in the epidemiologic record. The common ancestor with strain H091640090 probably existed in the recent past (≈20 years) because only ≈10 SNPs have accumulated in the genome since the lineages diverged, according to the short-term substitution rate (1–2 SNPs/genome/y) previously reported for *Salmonella* epidemics (*26*,*27*).

Because virtually all monophasic strains from the current epidemic clade encoded SGI-3 but isolates from outside the clade did not, initiation of clonal expansion was probably accompanied by the acquisition of this genomic island. SGI-3 encodes resistance to heavy metals, including copper and zinc, which is potentially relevant because these are supplements commonly added to pig feed as micronutrients and general antimicrobials (*28*). Indeed, in the European Union, heavy metals have been used increasingly in response to the ban on nonspecific use of antimicrobial drugs in animal feed for growth promotion (*29*). Concentration of heavy metals in pig intestines may represent substantial selective pressure contributing to the success of this clone. Indeed, a recent study reported that an enhanced MIC (20–24 mmol/L) compared with the baseline MIC (16 mmol/L) for copper sulfate was significantly more likely to be found in isolates from pig feces (*30*).

A remarkable feature of the monophasic *Salmonella* Typhimurium epidemic in the United Kingdom is the considerable number of polymorphisms that affect coding capacity that occurred during the short period (≈10–15 years) of clonal expansion of the epidemic clade. These include a complex pattern of deletions in the *fljB* locus and surrounding genome sequence, insertions in the *thrW* locus, and acquisition of a novel phage carrying the *sopE* gene. These polymorphisms seem to be stable and not deleterious because they all appear in parts of the tree that have subsequently undergone further clonal expansion. Deletions in the *fljB* locus that occurred subsequent to the initial clonal expansion of the epidemic clade accounted for the monophasic phenotype exhibited by most of these isolates. The high frequency of deletions in this locus may be the result of a composite Tn21-like transposable element that is inserted in the *hin*–*iroB* intergenic region, a well-known characteristic of such insertions (*31*).

The acquisition of the *sopE* gene on a novel prophage element that occurred through multiple recent independent events may strongly affect the pathogenesis and epidemiology of the current epidemic. Lysogeny by phages carrying the *sopE* gene has been associated with epidemic strains of *Salmonella* Typhimurium and of other *Salmonella* serotypes (*32*). The expression of SopE may increase the fitness of the pathogen, a possibility consistent with the observation that recent acquisition of the *sopE* gene by monophasic epidemic isolates has been followed by an increase in the frequency of *sopE*-positive isolates. The ability to induce inflammatory diarrhea is a main strategy for the transmission of *Salmonella* Typhimurium. SopE is a guanine exchange factor that activates both cdc42 and rac1; *sopE2* activates only cdc42 (*33*). All *Salmonella* Typhimurium strains sequenced to date encode the *sopE2* gene that exhibits 59% identity with SopE. The additional activity of SopE has a marked effect on the outcome of the interaction of *Salmonella* Typhimurium with the intestinal mucosa, resulting in increased amounts of salmonellae in the intestinal lumen and shedding in the feces. SopE expression results in increased production of host nitrate, a valuable electron acceptor used by *Salmonella* Typhimurium for respiration (*34*).

In conclusion, our findings indicate that the current monophasic *Salmonella* Typhimurium clone associated with many animal species and human clinical infections in the United Kingdom arose recently. Subsequent microevolution in a short time has resulted in considerable genotypic variation affecting antigens, virulence factors, and resistance loci. Some genomic features, such as resistance to heavy metals, may have resulted in initial selection for the current clone, while more recent horizontal gene transfer or deletions and plasmid loss may have generated variation selected during the epidemic.

**Technical Appendix 1.** Strains and metadata used in study of microevolution of monophasic *Salmonella* Typhimurium during an epidemic in the United Kingdom.

**Technical Appendix 2.** Additional drug resistance and genetic data for *Salmonella* Typhimurium and monophasic *Salmonella* Typhimurium isolated from humans, livestock, or contaminated food from the United Kingdom or Italy during 1993–2010.
