# Correction: Vol. 22, No. 4

**DOI:** 10.3201/eid2210.C12210

**Published:** 2016-10

**Keywords:** errata, erratum, correction

The authors of Microevolution of Monophasic *Salmonella* Typhimurium during Epidemic, United Kingdom, 2005–2010 (L. Petrovska al.) have provided the following Addendum: “It has come to the authors’ attention that the designation ‘*Salmonella* Genetic Island 3 (SGI-3)’ has been previously assigned to a 31-kb genomic island in a strain of *Salmonella* Mississippi (http://dx.doi.org/10.1371/journal.pone.0041247). To avoid confusion in the literature, we propose that the SGI-3 referred in our manuscript be designated SGI-4 in future reference.” In addition, some of the accession numbers listed in online Technical Appendix 1 were incorrect, and funding sources and author contact information for the article have also been updated. The article has been corrected online (http://wwwnc.cdc.gov/eid/article/22/4/15-0531_article).

