# Supplementary material for: Microevolution of Monophasic Salmonella Typhimurium during Epidemic, United Kingdom, 2005–2010
Source: Emerg Infect Dis. 2016 Apr;22(4):617–24. doi: 10.3201/eid2204.150531 (PMC4806966; doi:10.3201/eid2204.150531)
Supplement: Supplementary file 2 — Technical Appendix 2. Additional drug resistance and genetic data for Salmonella Typhimurium and monophasic Salmonella Typhimurium isolated from humans, livestock, or contaminated food from the United Kingdom or Italy during 1993–2010. [file 15-0531-Techapp-s2.pdf]

Article DOI: http://dx.doi.org/10.3201/eid2204.150531

Microevolution of Monophasic *Salmonella* Typhimurium during Epidemic, United Kingdom

Technical Appendix 2

Technical Appendix 2 Table. Additional strains and metadata, strains used to determine *sopE* frequency

Year of isolation	Sample name	Species	Serotype	Phage type	Resistance pattern	*SopE*, by PCR
2005	S04612-05	ENVIRONMENTAL_FARM	4,12:I:-	NOPT	ASSuT	Neg
2005	S07300-05	CATTLE	4,12:I:-	NOPT	ND	Neg
2006	S06813-06	PIG	TYPHIMURIUM	DT 193	ASSuT	Neg
2006	S00065-06	CATTLE	4,12:I:-	NOPT	ND	Neg
2007	L00745-07	PIG	4,5,12:I:-	NOPT	TASxtSSU	Pos
2007	S05720-07	CATTLE	TYPHIMURIUM	DT 193	ASSuT	Neg
2007	L00653-07	PIG	4,12:I:-	NOPT	ASSuT	Neg
2007	S00250-07	PIG	4,12:I:-	ND	Sensitive	Neg
2007	S04962-07	CATTLE	4,12:I:-	NOPT	ASSuT	Neg
2007	L00650-07	PIG	4,5,12:I:-	NOPT	T	Neg
2007	S06676-07	PIG	4,5,12:I:-	NOPT	ASSuT	Neg
2008	L00555-08	DUCK	4,12:I:-	ND	T	Neg
2008	L01189-08	PIG	4,12:I:-	ND	T	Neg
2008	S05635-08	OTHER_VEG_MINERAL	4,12:I:-	ND	ASSuT	Neg
2008	S06669-08	DOG	4,12:I:-	ND	ASSuT	Neg
2008	S06718-08	CATTLE	4,12:I:-	ND	ASSuT	Neg
2009	S04409-09	PIG	4,12:I:-	DT 193	ASSuT	Pos
2009	LO506-09	PIG	4,5,12:i:-	DT 193	ASSuNaCnApr	Pos
2009	L00028-09	PIG	4,12:I:-	DT 193	ASSuT	Neg
2009	L00041-09	DOG	4,12:I:-	DT 193	ASSuT	Neg
2009	L00300-09	DOG	4,12:I:-	DT 193	TA	Neg
2009	L00663-09	MOUSE	4,12:I:-	DT 191a	ASSuT	Neg
2009	S00176-09	PIG	4,12:I:-	RDNC	ASSuT	Neg
2009	S00428-09	DOG	4,12:I:-	DT 193	ASSuT	Neg
2009	S04117-09	SHEEP	4,12:I:-	DT 193	ASSu	Neg
2009	S04700-09	CAT	4,12:I:-	DT 193	TASxtSSu	Neg
2009	S04711-09	DOG	4,12:I:-	DT 193	ASSuT	Neg
2010	S02497-10	EQ_HORSE	4,12:I:-	DT 193	ASSuT	Pos
2010	S03660-10	CHICKEN	4,5,12:I:-	ND	Sensitive	Pos
2010	S00028-10	CATTLE	4,12:I:-	DT 193	ASSuT	Neg
2010	S00474-10	CAT	4,12:I:-	DT 193	ASSuT	Neg
2010	S00814-10	CHICKEN	4,12:I:-	DT 193	ASSuT	Neg
2010	S01299-10	PIG	4,12:I:-	DT 193	ASSuT	Neg
2010	S01332-10	CAT	4,12:I:-	DT 193	ASSuT	Neg
2010	S01585-10	OTHER_VEG_MINERAL	4,12:I:-	DT 193	TNASxtSSu	Neg
2010	S01764-10	CHICKEN	4,12:I:-	DT 193	ASSuT	Neg
2010	S03060-10	CATTLE	4,12:I:-	DT 193	T	Neg
2010	L00809-10	PIG	4,5,12:I:-	DT 193	ASSuT	Neg
2010	L01001-10	CHICKEN	4,5,12:I:-	DT 120	ASSuT	Neg
2010	S00771-10	PIG	4,5,12:I:-	DT 193	TNASxtCCnSSuApr	Neg

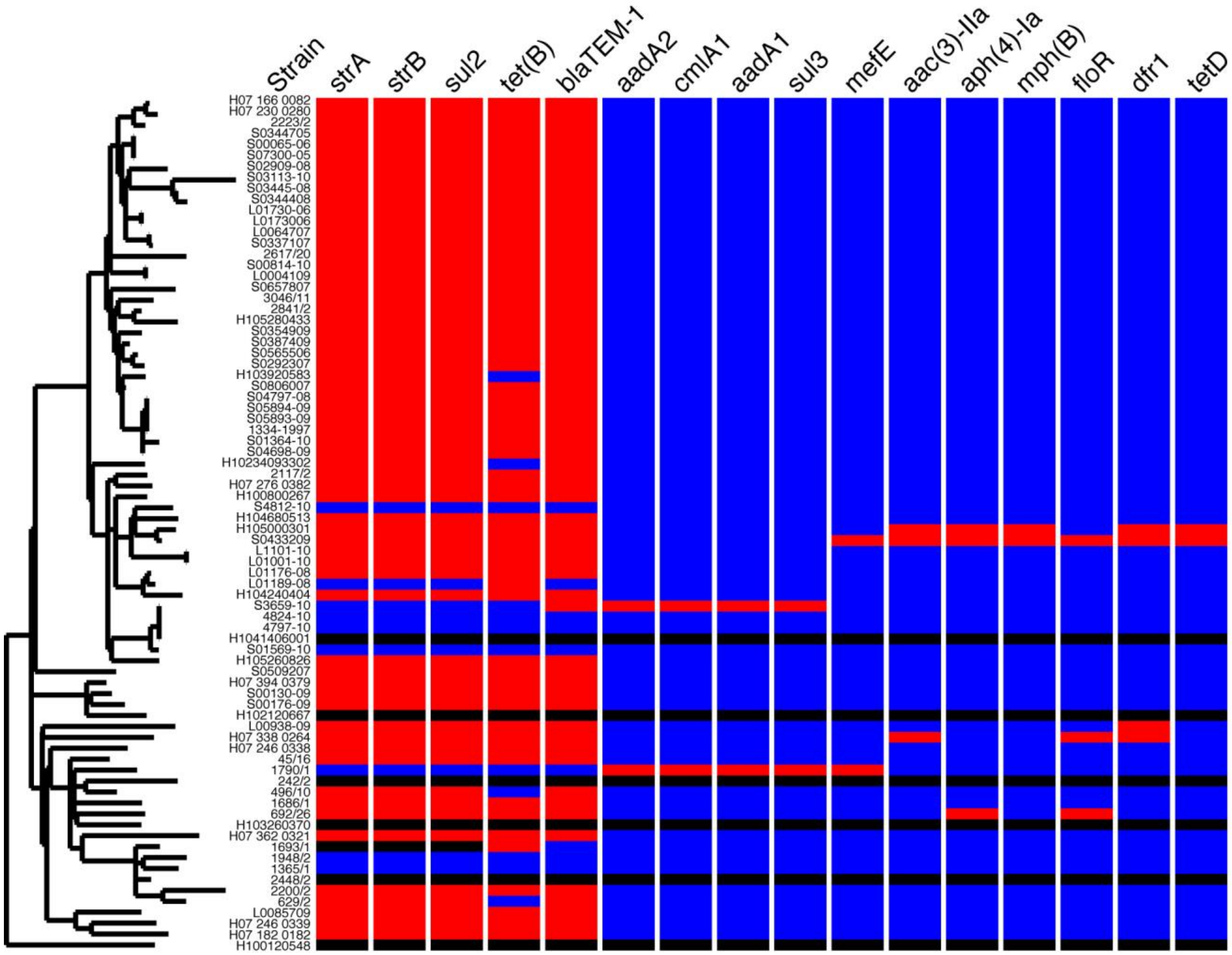

Technical Appendix 2 Figure 1. Presence of antibiotic resistance genes in the monophasic *Salmonella* Typhimurium epidemic strains from the UK. The presence (red) or absence (blue) of antibiotic resistance genes are shown in the context of the maximum likelihood tree described in Figure 2 in the main text. Some data were unavailable due to poor quality sequence assembly (black).

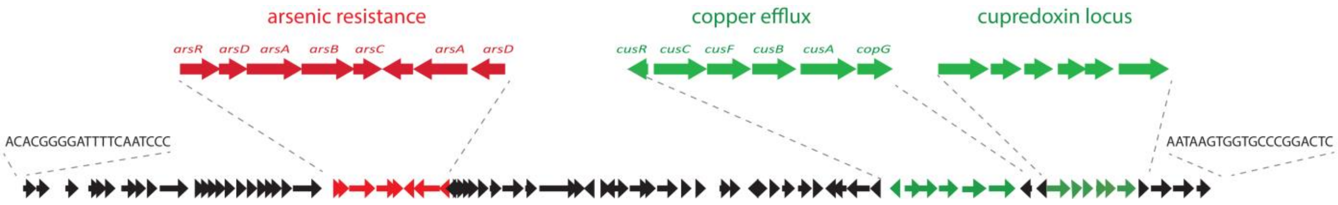

Technical Appendix 2 Figure 2. Gene arrangement of the novel genomic island of *Salmonella* 1,4,[5],12:i:- strain SO4698–09. Arrows indicate predicted genes within the island. The position of genes with predicted functions by sequence comparison are indicated for arsenic resistance (red), cadmium, zinc and copper resistance (green). The nucleotide sequence flanking the insertion in the whole genome sequence of SO4698–09 (PRJEB10340) is indicated.

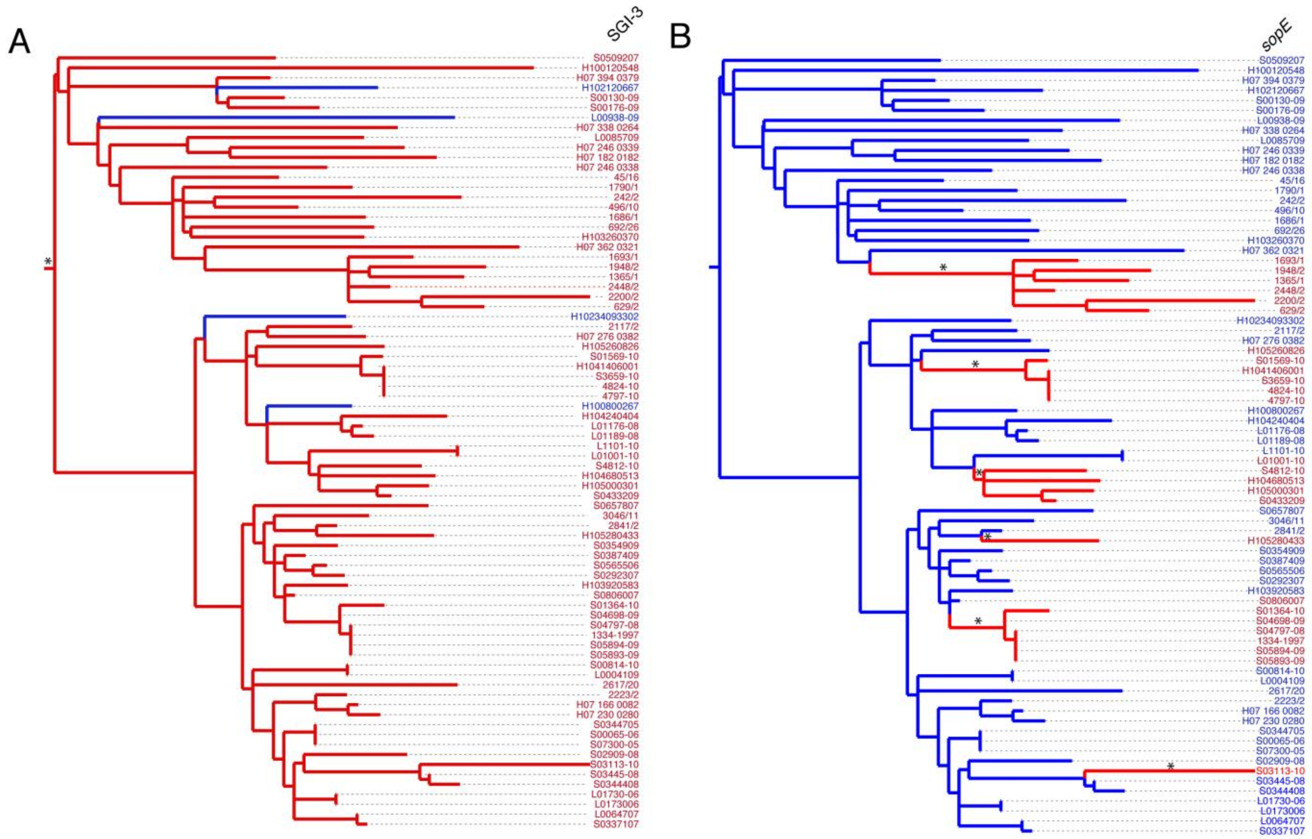

Technical Appendix 2 Figure 3. Ancestral state reconstruction of SGI-3 and *sopE* gene within the monophasic epidemic clade. Maximum likelihood trees for 77 UK and Italy monophasic isolates as previously described in Figure 2 in the main text. Ancestral state for presence (red edges) or absence (blue edges) of SGI-3 (A) or *sopE* (B) were reconstructed based on maximum parsimony using ACCTRAN. * indicate the inferred acquisition of the genetic element.

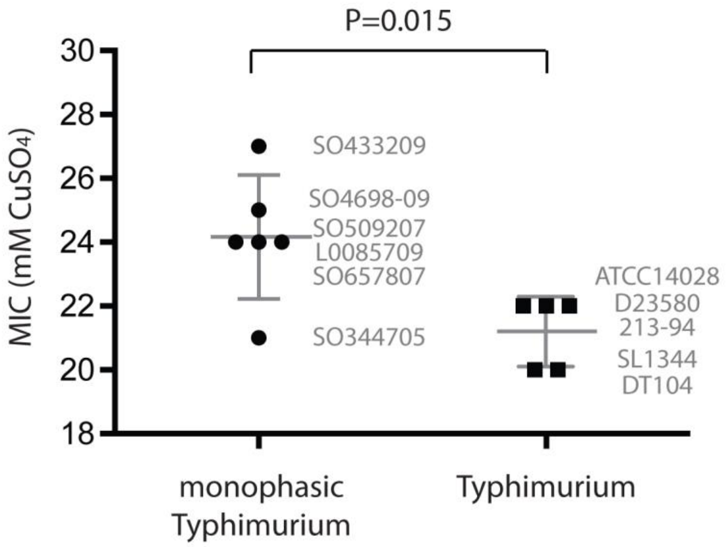

Technical Appendix 2 Figure 4. MIC of monophasic *Salmonella* Typhimurium and *Salmonella* Typhimurium isolates to copper sulfate in rich broth culture. The ability of monophasic *Salmonella* Typhimurium (filled circles) or *Salmonella* Typhimurium (filled squares) isolates to grow in Luria Bertani broth in the presence of copper sulfate (pH7) were monitored by the optical density of culture. The MIC was defined as the concentration at which cultures attained at least OD_{600nm} of 0.1. The mean for each phylogenetic group (gray bar) +/– standard deviation are indicated. Student's t test was used to test significance.

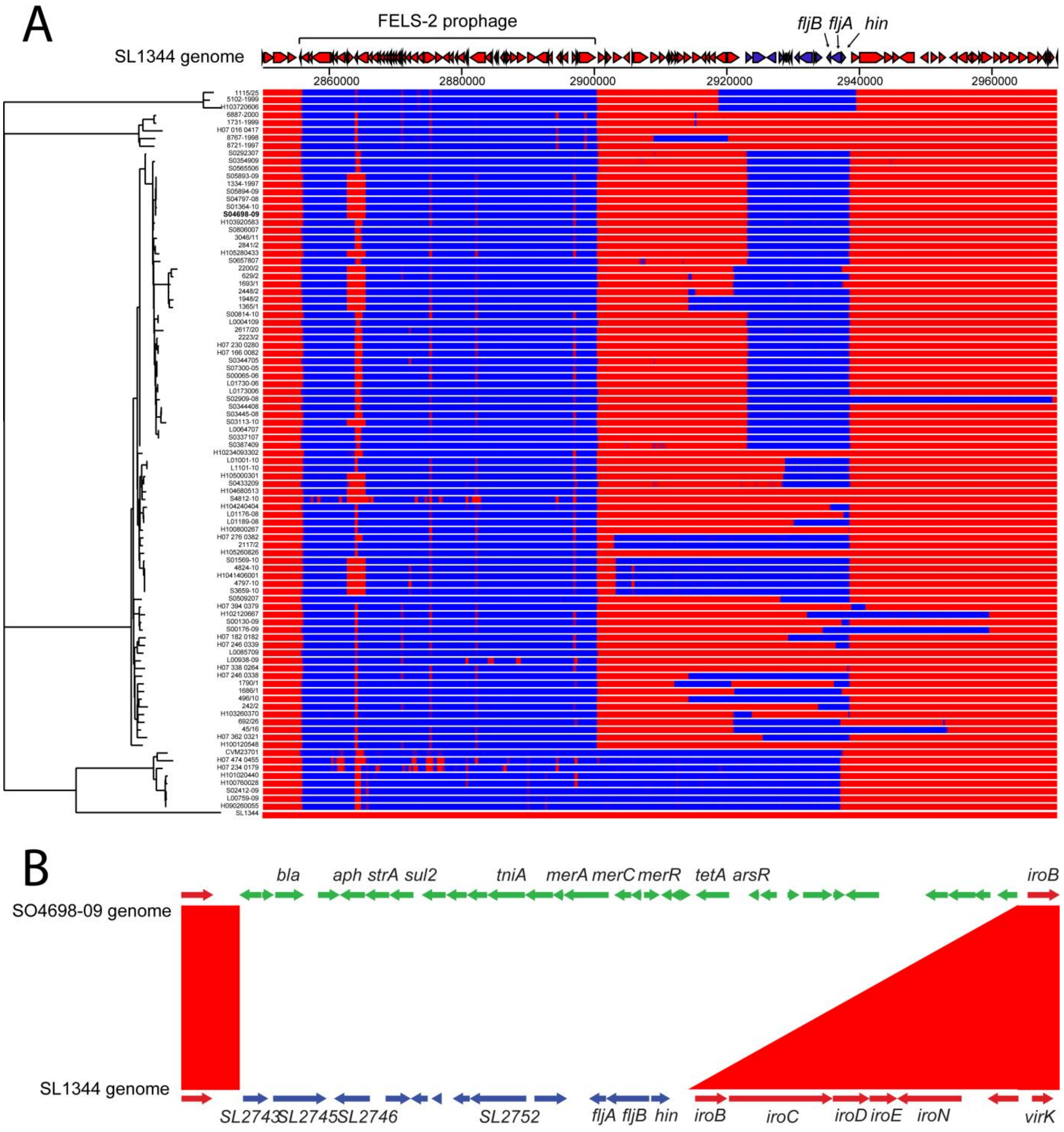

Technical Appendix 2 Figure 5. Heat map showing deletions around the *fljB* locus of the *Salmonella* 1,4,[5],12:i:- epidemic clade isolates. The heat map (A) indicating mapped sequence read coverage for *Salmonella* 1,4,[5],12:i:- epidemic clade isolates to the *fljB* locus and flanking sequence of the whole genome sequence of *Salmonella* Typhimurium strain SL1344. Color indicates 0 mapped reads (blue) to ≥20 bases (red). Filled arrows indicate genes in the SL1344 genome sequence as described previously (*1*). A maximum likelihood tree of phenotypically monophasic isolates from the strain collection is shown.

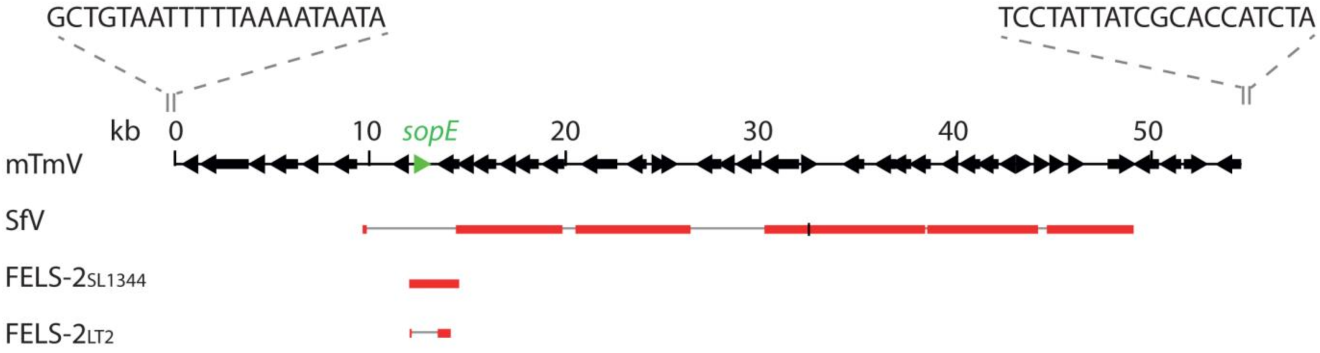

Technical Appendix 2 Figure 6. Prophage element mTmV from strain SO4698–09 and BLAST results with SfV and FELS-2 prophage. Predicted open reading frames in the 55 kb mTmV prophage of strain SO4698 are shown with flanking nucleotide sequence for orientation. Regions with significant BLAST results (red bar) in the related prophage SfV prophage and FELS-2 prophages are indicated below.

Reference

1. Kröger C, Dillon SC, Cameron AD, Papenfort K, Sivasankaran SK, Hokamp K, et al. The transcriptional landscape and small RNAs of *Salmonella enterica* serovar Typhimurium. Proc Natl Acad Sci U S A. 2012;109:E1277–86. PubMed http://dx.doi.org/10.1073/pnas.1201061109
